# Maternal Transmission of a Humanised *Igf2r* Allele Results in an *Igf2* Dependent Hypomorphic and Non-Viable Growth Phenotype

**DOI:** 10.1371/journal.pone.0057270

**Published:** 2013-02-28

**Authors:** Jennifer Hughes, Susana Frago, Claudia Bühnemann, Emma J. Carter, A. Bassim Hassan

**Affiliations:** Cancer Research UK Tumour Growth Group, Oxford Molecular Pathology Institute, Sir William Dunn School of Pathology, University of Oxford, Oxford, United Kingdom; CNRS, France

## Abstract

The cation independent mannose 6-phosphate/insulin-like growth factor 2 receptor (IGF2R) functions in the transportation and regulation of insulin-like growth factor 2 (IGF2) and mannose 6-phosphate modified proteins. The relative and specific titration of IGF2 by high affinity binding of IGF2R represents a mechanism that supports the parental conflict theory of genomic imprinting. Imprinting of *Igf2* (paternal allele expressed) and *Igf2r* (maternal allele expressed) arose to regulate the relative supply of both proteins. Experiments in the mouse have established that loss of the maternal allele of *Igf2r* results in disproportionate growth and peri-natal lethality. In order to systematically investigate the consequences of loss of function and of hypomorphic alleles of *Igf2r* on growth functions, we introduced a conditional human *IGF2R* exon 3–48 cDNA into the intron 2 region of murine *Igf2r*. Here we show that the knock-in construct resulted in over-growth when the humanised *Igf2r* allele was maternally transmitted, a phenotype that was rescued by either paternal transmission of the humanised allele, expression of a wild-type paternal allele or loss of function of *Igf2*. We also show that expression of IGF2R protein was reduced to less than 50% overall in tissues previously known to be *Igf2* growth dependent. This occurred despite the detection of mouse derived peptides, suggesting that trans-splicing of the knock-in human cDNA with the endogenous maternal mouse *Igf2r* allele. The phenotype following maternal transmission of the humanised allele resulted in overgrowth of the embryo, heart and placenta with partial peri-natal lethality, suggesting that further generation of hypomorphic *Igf2r* alleles are likely to be at the borderline of maintaining *Igf2* dependent viability.

## Introduction

Genomic imprinting in mammals results in silencing of one of the parental alleles of a gene. At least 60 mammalian imprinted genes have been discovered, many of which regulate embryonic growth and development [1]. The function of the cation independent mannose 6-phosphate/insulin-like growth factor 2 receptor (IGF2R) is in the transportation and regulation of the extra-cellular bioavailability of Insulin-like growth factor 2 (IGF2) and mannose 6-phosphate modified proteins [2]. *Igf2r* is imprinted in post-implantation tissues in the mouse, with maternal allele expression up-regulated relative to expression of the paternal allele [3]. Differential allele expression is dependent on methylation of the maternal promoter of an anti-sense long non-coding RNA (ncRNA) called *Airn* located within intron 2 of the *Igf2r* gene [3]. Disruption of the promoter, deletion or truncation of *Airn* results in abrogation of the anti-sense transcript, and establishment of relative paternal allele *Igf2r* expression similar to the maternal allele (bi-allelic) [4], [5], [6], [7]. The mechanism by which the titration of *Airn* up-regulates maternal allelic expression up to ten-fold appears to be independent of expression from its own promoter, but results in *Igf2r* paternal promoter methylation [3], [5]. Gain of function of *Igf2r* following region 2 deletion within the paternal *Igf2r* allele results in bi-allelic expression, leading to an *Igf2* dependent growth reduction in embryos and placenta [7]. Gain of function through transgene expression of IGF2R also results in *Igf2* dependent reduction in organ growth [8], [9], [10], [11]. Conversely, loss of function of the maternal allele of *Igf2r* results in *Igf2* dependent disproportionate embryonic, heart, and placental overgrowth and is associated with peri-natal lethality [12], [13], [14], [15]. These data suggest that gene dosage of *Igf2r* is able to titrate the supply of *Igf2* to regulate mammalian growth, and that there is a dependency on maternal allele expression for survival.

The relative and specific titration of IGF2 by high affinity binding of IGF2R represents a mechanism that supports the parental conflict theory. Here resources transmitted to the developing conceptus are a result of competition between males attempting to extract resources for their offspring from mothers attempting to conserve such resources [16], [17]. The implication is that genomic imprinting of *Igf2* (paternal allele expressed) and *Igf2r* (maternal allele expressed) arose to regulate the relative supply of both proteins. The titration of the functional supply of IGF2 in this case would then be regulated at the gene expression level, whilst the overall efficiency of IGF2R as an IGF2 protein antagonist might be further regulated either at a tissue specific level or by post-transcriptional processes. Experiments described above in the mouse have established that loss of the maternal allele of *Igf2r* appears to result in disproportionate overgrowth and lethality and appears to offer no growth or survival advantage. Similar observations have followed mammalian somatic cell cloning that may also account for the ‘large offspring syndrome’ phenotype particularly in sheep [18]. Bi-allelic expression of *Igf2* is however associated with proportionate growth, with the potential for the viable phenotypes to undergo evolutionary selection for size, presumably because the capacity of *Igf2r* remains unsaturated and is still able to limit disproportionate overgrowth [19]. Between these extremes it remains unknown what level of titration of the maternal expressed *Igf2r* (or hypomorphic allele) can retain viability through IGF2 supply regulation. It is also unknown whether mono-allelic *Igf2r* expression, with the associated high affinity binding for IGF2, already represents the minimal evolutionary state selected for *Igf2r* to limit disproportionate mammalian growth and promote viability.

In view of the strict dependency on a single expressed allele of *Igf2r* for viability, modification by loss of function to generate domain specific, either complete or partial loss of function alleles (hypomorphic alleles), such that may arise from mutations, splicing de-regulation, mRNA stability, siRNA and post-translational processing, may reveal the relative contributions of these functions to growth. For example, we lack models to investigate germ-line non-synonymous *IGF2R* polymorphisms that occur in humans and the somatic loss of function mutations that detected in common human cancers, with the latter frequently co-existing with loss of heterozygosity of *IGF2R*. [20], [21], [22], [23], [24], [25], [26]. Gain of function mutations have also been detected during the establishment and subsequent evolution of the acquisition of IGF2 binding to domain 11 of *IGF2R* [27]. With 48 exons spaced over 120kb, efficient splicing and mRNA processing are also likely to be essential for IGF2R production that may have co-evolved with *Igf2* imprinting [27].

In order to begin to investigate the consequences of loss of function alleles on *Igf2r* growth functions, we first introduced a conditional human *IGF2R* exon 3–48 cDNA into the intron 2 region of murine *Igf2r*. We assumed that this would functionally complement the endogenous wild-type mouse allele via splicing of exon 2 of the endogenous mouse gene to the inserted cDNA. Here we show that the knock-in humanised construct resulted in an over-growth phenotype when the humanised allele was maternally transmitted (HUm = humanised maternal allele, HUp = humanised paternal allele), and that this phenotype could be genetically rescued. We show that in HUm mice the level of IGF2R protein was reduced overall in tissues previously known to be *Igf2* growth dependent, despite the additional detection of co-existing peptides derived from mouse protein, suggesting that trans-splicing of the knock-in human cDNA occurred with the endogenous maternal mouse *Igf2r* allele. The phenotype of the hypomorphic humanised allele resulted in overgrowth of the embryo heart and placenta with partial peri-natal lethality, suggesting that this particular humanised hypomorphic loss of function allele was at the borderline of maintaining viability.

## Results

**Figure 1:**
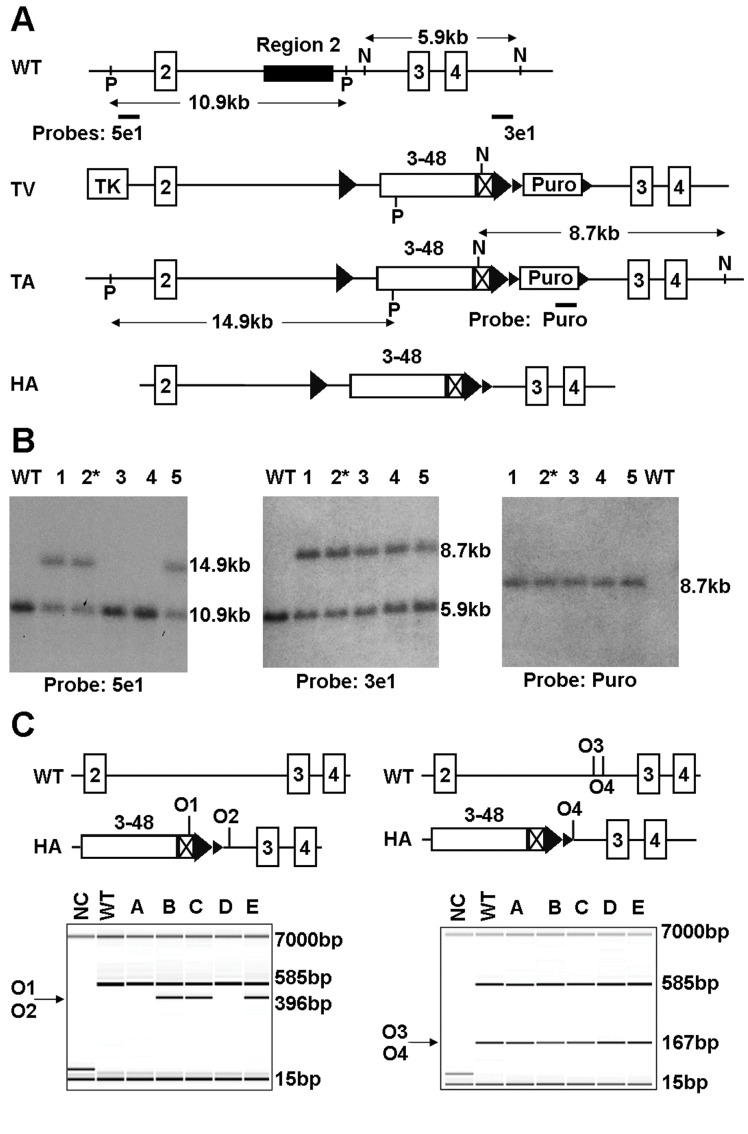
Transgene targeting and genotyping. (**A**) Schematic diagram of *Igf2r* humanisation targeting strategy. Exons are represented as numbered boxes, *lox*P sites as small triangles, FRT sites as large triangles, human IGF2R mini-gene (Exons 3–48) as open box, mouse 3′ UTR as crossed box. WT, wild type; TV, targeting vector; TA, targeted allele; HA, humanised allele; P, PspOMI; N, NsiI; O1, oligo1; O2, oligo 2; O3, oligo 3; O4, oligo 4. (**B**) To detect correct homologous recombination at 5′, 5 clones of ES cells were digested with PspOMI and hybridised to probe 5e1 (left panel), 3 clones gave the expected band at 14.9 kb. For the 3′flank, cells were digested with NsiI and hybridised to probe 3e1 (central panel), all 5 gave the expected band at 8.7 kb. To detect single insertion, the clones were digested with NsiI and hybridised to probe Puro and all 5 gave a single band at the correct size of 8.7 kb (right panel). * denotes clone 2 chosen for breeding (A-D09). (**C**) Genotyping analysis was carried out by PCR on pups bred from clone 2 in (**B**). (Left panel) The 396 bp fragment (arrow) amplified by oligos 1 and 2 denotes the presence of heterozygous/homozygous humanised alleles. Wild type allele is not detected. (Right panel) The 167 bp fragment (arrow) amplified by oligos 3 and 4 denotes the presence of heterozygous and homozygous wild type alleles. In both PCR reactions, an internal control fragment of 585 bp was included. Marker bands of 15 bp and 7000 bp are shown. NC, negative control.

To generate a humanised allele of mouse *Igf2r*, we targeted the human IGF2R exons 3–48 cDNA into the intron 2 region by homologous recombination (Fig. 1). The targeting vector replaced the intron 2 *Airn* promoter region but retained the 3′ splice site of mouse exon 2, with the 5′ splice site of human exon 3 distal to a flp site flanked human cDNA and mouse derived 3′UTR. C57BL6 ES lines were transformed under positive (puromycin) and negative selection with gancyclovir. Following PCR verification of correct targeting, and Southern blot to confirm a single copy insert, two lines were bred with a Cre-deleter strain to remove the puromycin selection cassette but retaining the flp sites. Two lines achieved germ-line transmission, with line 2* being the basis of the experimental breeding and phenotyping (Fig. 1B and C).

**Figure 2:**
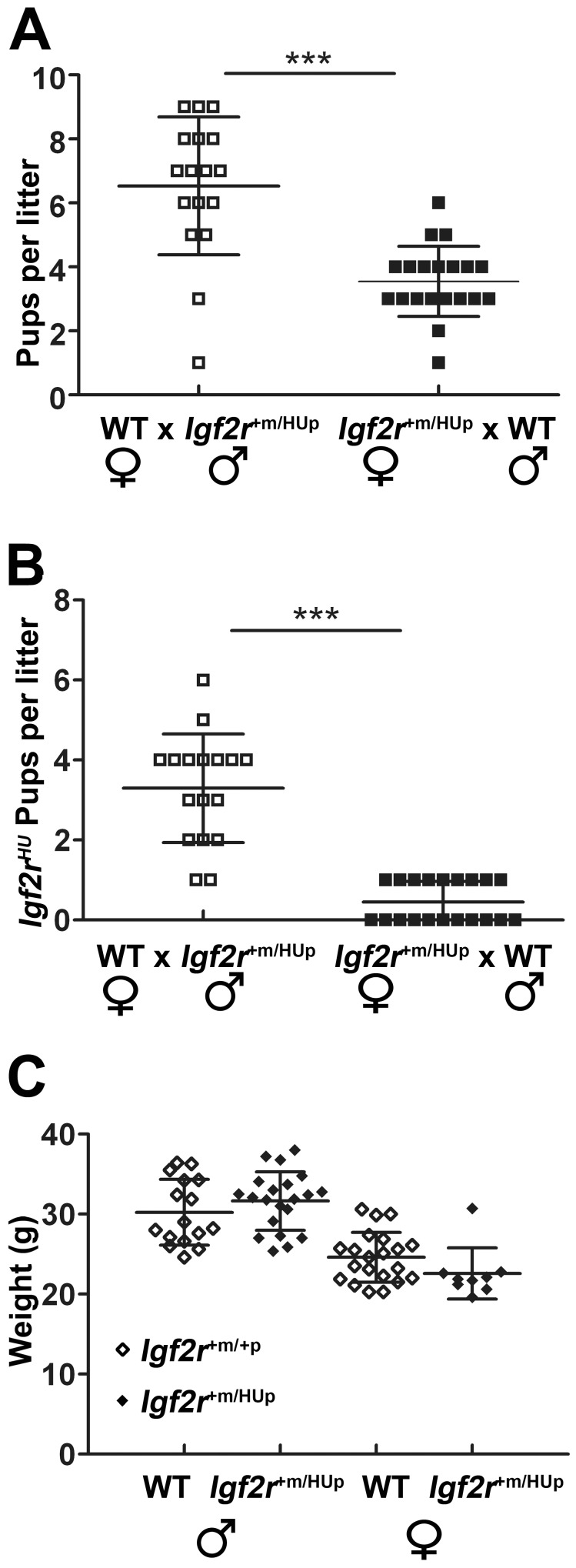
Maternal and paternal transmission of *Igf2r^+m/HU.p^* (**A**) Total number of neonates per litter at postnatal day 10 resulting from either paternal transmission (wild-type females crossed to *Igf2r^+m/HUp^* males, 8 pairs, 20 litters, open squares) or maternal transmission (*Igf2r^+m/HUp^* females crossed to wild-type males, 4 pairs, 17 litters, closed squares). Litter sizes were significantly reduced following maternal transmission. (**B**) Number of *Igf2r^+m^/^HUp^* pups present at postnatal day 10 in the litters described in (**A**) indicating that maternal transmission results reduced numbers of pups with the humanised allele. (**C**) Adult body weights (measured at 10 weeks) of offspring resulting from paternal transmission where there was no associated lethality (wild-type females crossed to *Igf2r^+m/HUp^* males, progeny; *Igf2r^+m/HUp^* males n = 16, wild-type males n = 21, *Igf2r^+m/HUp^* females n = 21, wild-type females n = 9). ***p<0.0001. Whiskers ± standard deviation. Paired t-test.

Maternal transmission of the humanised allele (HUm) resulted in reduced litter sizes compared to paternal transmission (HUp) (Fig. 2A). Genotyping with allele specific primers revealed that very few *Igf2r^HUm/+p^* pups survived following maternal transmission with wild-type males (Table 1). Examination of twenty litters from eight breeding pairs resulted in only nine viable *Igf2r^HUm/+p^* progeny compared to sixty two *Igf2r^+m/+p^* littermate controls (χ^2^, P<0.0001) (Fig. 2B and Table 1). Following paternal transmission, all pups were viable and *Igf2r^+m/HUp^* embryos were the same as wild-type littermates in weight (Table 1 and Fig. 2C).

**Table 1:** Summary of breeding outcomes following humanised *lgf2r* allele transmission.

Humanised *Igf2r*allele Transmission	Summary of breeding outcomes				
	Cross	Genotypes	Observed	Expected	
Maternal	*Igf2r^+m/HUp^*♀×WT♂(20 litters, 8 pairs)	*Igf2r* ^+m/+p^ *Igf2r* ^HUm/+p^	629	35.535.5	P<0.0001*X* ^2^ = 39.563
Paternal	WT♀×*Igf2r^+m/HUp^*♂ (17 litters, 4 pairs)	*Igf2r* ^+m/+p^	55	55.5	P = NS
		*Igf2r* ^+m/HUp^	56	55.5	
Homozygote	*Igf2r^+m/HUp^*♀×*Igf2r^+m/HUp^*♂(25 litters, 6 pairs)	*Igf2r* ^+m/+p^	39	32.75	P = NS
		*Igf2r* ^HUm/+p^ *Or Igf2r* ^+m/HUp^	74	65.5	
		*Igf2r* ^HUm/HUp^	18	32.75	
Maternal	*Igf2r^+m/HUp^*♀×*Igf2* ^+/−^♂ (11 litters 4 pairs)	*Igf2* ^+/+^, *Igf2r* ^+/+^	17	15.25	P = NS*
		*Igf2* ^+m/−p^, *Igf2r* ^+m/+p^	16	15.25	
		*Igf2* ^+m/+p^, *Igf2r* ^HUm/+p^	8	15.25	
		*Igf2* ^+m/−p^, *Igf2r* ^HUm/+p^	20	15.25	
Maternal	*Igf2r^+m/HUp^*♀×*R2Δ^+m/−p^*♂, (6 litters, 2 pairs)	*R2 Δ* ^ +m/−p^, *Igf2r* ^+m/+p^	23	24.5	P = NS
		*R2 Δ* ^ +m/−p^, *Igf2r* ^HUm/+p^	26	24.5	

* *X* ^2^ and One way ANOVA, Kruskal-Wallis, with Dunns multiple comparison post-test.

**Figure 3:**
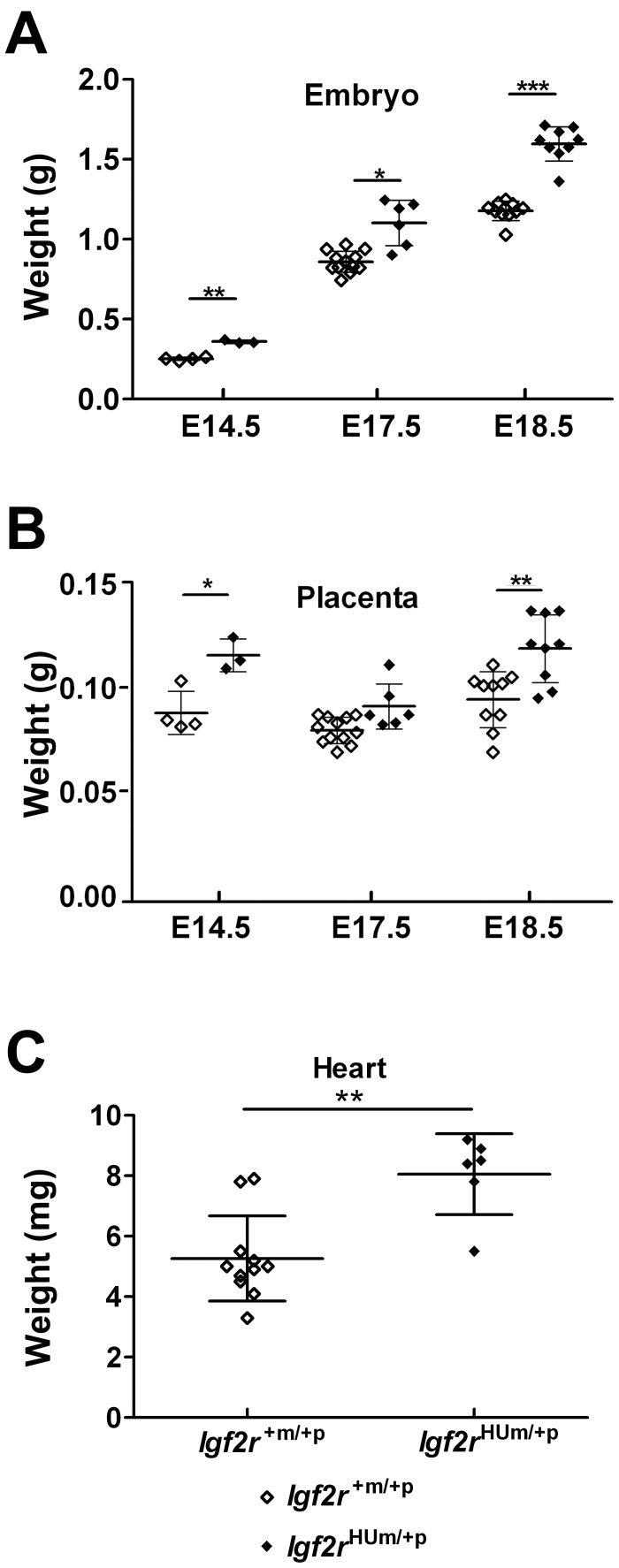
Embryonic, placental and heart growth following maternal transmission of *Igf2r^+m/HUp^.* (**A**) Total body weights at E14.5 (WT n = 4, *Igf2r^HUm/+p^* n = 3), E17.5 (WT n = 12, *Igf2r^HUm/+p^* n = 6) and E18.5 (WT n = 10, *Igf2r* ^HUm/+p^ n = 9) showing gain in weight of *Igf2r* ^HUm/+p^. (**B**) Placental weights at E14.5 (WT n = 4, *Igf2r^HUm/+p^* n = 3) E17.5 (WT n = 12, *Igf2r^HUm/+p^* n = 6) and E18.5 (WT n = 10, *Igf2r^HUm/+p^* n = 9). At E14.5 P = 0.0148, at E17.5 P = NS, at E18.5 P = 0.0037. (**C**) Heart weights at E18.5 (WT n = 11, *Igf2r^HUm/+p^* n = 6) showing gain in weight of hearts of *Igf2r* ^HUm/+p^. *P<0.05, **P<0.01, ***P<0.001. Whiskers ± standard deviation. Paired t-test.

Previously reported germline *Igf2r* loss of function identified a placental and embryonic overgrowth (120–135%) phenotype starting around E12.5 [12], [13], [14]. We analysed embryonic growth following timed matings using maternal transmission of the humanised allele. By E14.5, *Igf2r^HUm/+p^* embryos, placentae and hearts were all significantly heavier than littermate controls with the relative overgrowth in the range of 105–120% at each time point (Fig. 3A, B and C). By E18.5, the ratio of heart to overall body weight was approximately 0.0073 in *Igf2r^HUm/+p^* embryos compared to 0.006 in *Igf2r^+m/+p^* littermate controls (not shown, NS), suggesting that the heart growth phenotype was not grossly disproportionate.

**Figure 4:**
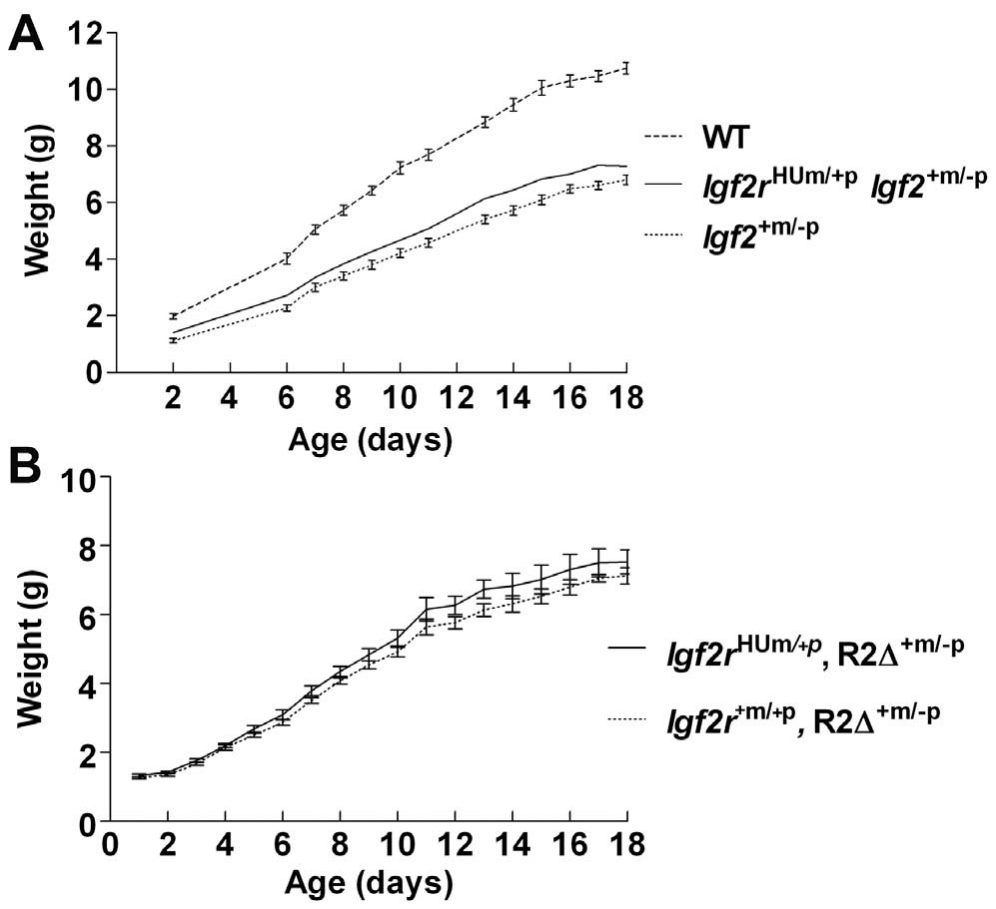
Rescue of *Igf2r^+m/HUp^*maternal transmission growth phenotype by loss of function of *Igf2* and gain of function of *Igf2r.* (**A**) Maternal transmission to generate *Igf2r^HUm/+p^* was combined with paternal transmission of the paternal null allele of *Igf2^+m/−p^* to generate combined heterozygotes (*Igf2r^HUm/+p^*, *Igf2^+m/−p^*). Post-natal growth curves show that maternal transmission of the maternal allele did not alter growth rates when lethality was rescued by loss of *Igf2* (Table 1). (**B**) Lethality of *Igf2r^HUm/+p^* was rescued by paternal expression of *Igf2r* murine allele (*R2Δ^+m/−p^*, see Table 1) and subsequent post-natal growth kinetics appeared similar.

**Figure 5:**
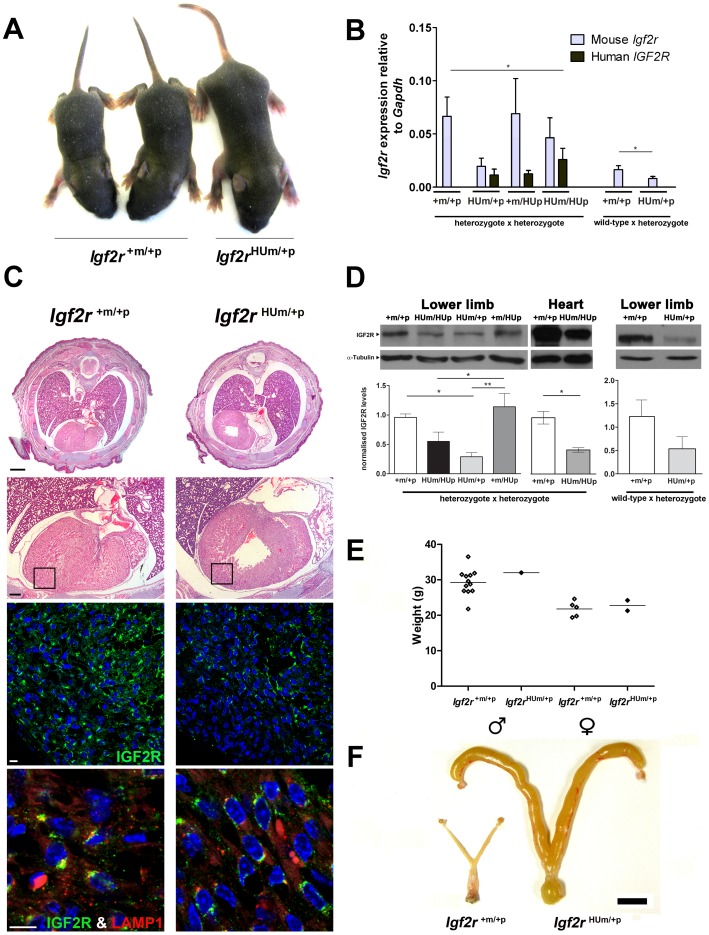
Embryonic heart IGF2R protein levels following maternal transmission of *Igf2r^+m/HUp^* (**A**) Two wild-type and a surviving *Igf2r^HUm/+p^* littermate at post-natal day 7. (B) Q-RT-PCR of *Igf2r* mRNA expression using mouse and human allele specific primers. Results are shown for progeny of heterozygote inter-cross to generate homozygote humanised knock-in alleles, compared to heterozygote progeny derived from maternal transmission. (*P<0.05, t-test, **P<0.01, 1 way ANOVA, Dunn’s post-test). Note, origin of the humanised allele in the heterozygote progeny was detected by exon 2–3 human and mouse specific RT-PCR. (**C**) Upper panel; H and E stained example of whole mouse embryo (E18.5) cross-sections below the level of the tracheal bifurcation showing dilated and enlarged heart in Hum. (Bar 1mm). Lower panels; higher power H and E images (Bar 0.25mm) showing regions visualised by immuno-fluoresence labelling of IGF2R (green), LAMP1 (red, for endosomal compartment localisation) and DAPI (nuclear DNA). Relative IGF2R levels appear reduced including the pericardial regions. (Bar 0.1mm). (D)Western blots of IGF2R in mouse embryos at day E18.5 in the lower limb and heart (n = 2–4). IGF2R levels were normalised to α-tubulin. Levels of IGF2R were reduced in limbs and hearts of embryos with a humanised maternal allele (HUm) derived from a heterozygous inter-cross (one way ANOVA with Tukey’s post-test, **P<0.005, *P<0.05), Levels of protein following maternal allele transmission alone also appear reduced but were not significant (n = 2). (E) Adult body weights (measured at 10 weeks) of surviving offspring resulting from maternal transmission of the humanised allele (*Igf2r^+m/HUp^* females crossed to wild-type males). Wild-type males n = 12 *Igf2r^HUm/+p^* males n = 1 wild-type females n = 5 *Igf2r^HUm/+p^* females n = 2. (C) Adult uteri from wild-type littermate and *Igf2r^Hum/+p^* surviving female showing dilated, enlarged and fluid filled uterine horns (scale bar = 1cm).

Combining the maternal transmitted allele with disruption of the paternal expressed allele of *Igf2* (*Igf2^+m/−p^*, [28]) resulted in rescue of the peri-natal lethality (Table 1). The surviving pups exhibited post-natal growth identical to *Igf2^+m/−p^*, i.e. similar kinetics to wild-type but at 60% of the weight (Fig. 4A). Combining the maternal transmitted allele with disruption of the intron 2 region of the paternal allele of *Igf2r* (*R2Δ^+m/−p^*, [7]) also resulted in rescue of the expected peri-natal lethality (Table 1). The surviving pups also exhibited similar post-natal growth to *R2Δ^+m/−p^* (Fig. 4B). These results indicate that the overgrowth phenotype and perinatal lethality were related to the supply of IGF2, and that expression of the murine paternal allele following imprinting disruption was sufficient to rescue the humanised maternal allele. Moreover, in the combined alleles (*R2Δ^+m/−p^*, *Igf2r^HUm/+p^*) disruption of *Airn* in intron 2 would have occurred in both alleles, indicating that the humanised allele was probably functioning as a hypomorphic allele independent of *Airn*. However, when we generated mice homozygous for the humanised allele (*Igf2r^HUmHUp^*), we surprisingly observed complete rescue of the post-natal over-growth and mortality phenotype (Table 1). From these latter data, we suggest that the function of a single humanised allele during spermatogenesis may have resulted in an overall reduction in expression of the *Airn* transcript, potentially resulting in ‘trans’ effects on the paternal transmitted wild-type allele, up-regulating the expression of paternal *Igf2r* allele (bi-allelic) expression, and therefore compensating for the hypomorphic function of the humanised maternal allele. Overall, there was a partial lethality phenotype associated with over-growth in *Igf2r^HUm/+p^*, suggesting that the humanised allele must be less than 50% of the normal wild-type function of a maternal expressed mouse allele of *Igf2r* (Fig. 5D).

**Figure 6:**
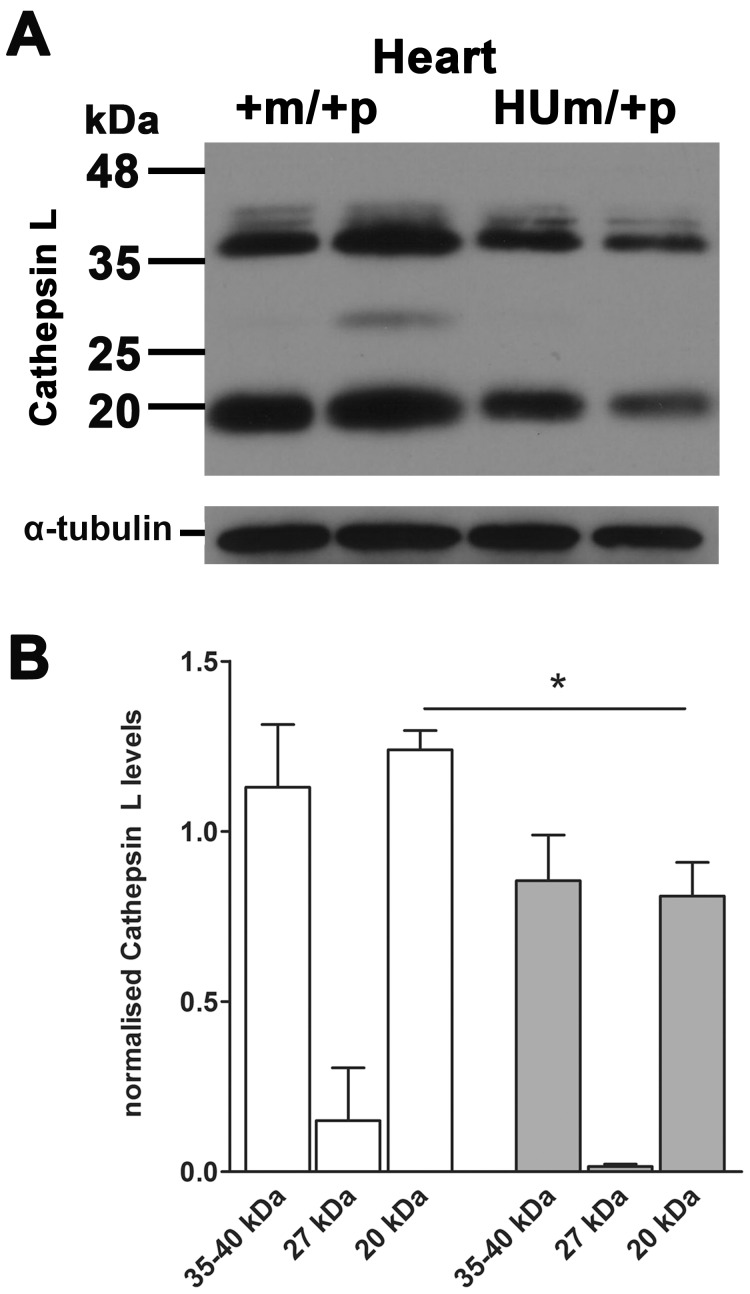
Embryonic heart Cathepsin L protein levels following maternal transmission of *Igf2r^+m/HUp^* (**A**) Western blots of Cathepsin L in mouse embryos at day E18.5. (**B**) Densitometry of protein levels normalised to α -tubulin loading control. Levels of cathepsin L were reduced in hearts of embryos with a humanised maternal allele (HUm) (P<0.05).

In order to investigate the cause of the reduced function of the humanised allele, we next performed quantitative RT-PCR to quantify gene expression (Fig 5B). Maternal transmission of the humanised allele following breeding with a wild-type male resulted in reduced mouse transcript levels compared to wild-type littermate controls. However, when transmitted by both females and males to generate litters containing homozygote humanised alleles, the relative mouse transcript levels appeared higher in all genotypes, supporting the potential for relaxation of paternal allele imprinting. The relative levels of the humanised alleles were higher in homozygotes as expected, but surprisingly, were expressed in addition with mouse transcripts (Fig. 5B). Analysis of heart tissue showed enlargement of the heart with an apparent increase in ventricular muscle (Fig. 5C). Morphometry of serial 5 µM sections of the hearts of E18.5 embryos stained with H&E revealed relatively dilated hearts with increased blood filled spaces within muscle (proportion of muscle to blood filled spaces, *Igf2r^+m/+p^* = 0.87, n = 3, *Igf2r^HUm/+p^* = 0.73, n = 4, p<0.01) and no evidence for hypertrophy based on the ratio of nuclei to heart muscle (ratio of nuclear to muscle area *Igf2r^+m/+p = ^*0.12 n = 3, *Igf2r^HUm/+p^* = 0.14 n = 4, NS). Immuno-localisation of IGF2R suggested reduced labelling throughout the heart, including the pericardium, but without gross change in IGF2R cytoplasmic distribution relative to an endosomal marker, LAMP1 (Fig. 5C). We next evaluated the levels of detectable IGF2R using western blots in the limbs and heart of embryos with heterozygote and homozygote humanised alleles. In *Igf2r^HUm/+p^* there was significantly less than 50% of the amount of IGF2R compared to WT control littermates, and close to 50% levels in homozygote *Igf2r^HUm/HUp^* mice, consistent with the genetic findings (Fig. 5D). These data suggest that the cause of the over-growth phenotype associated with humanisation of the murine *Igf2r* allele was the reduced IGF2R protein levels (approximately 30% of controls). We failed to detect increased levels of soluble IGF2 in either serum or heart, although increased low molecular weight forms of IGF2 were detected in embryonic limbs in *Igf2r^HUm/+p^* (not shown). We also screened a number of lysosomal proteases, and detected reduced levels of cathepsin L in heart muscle of *Igf2r^HUm/+p^* embryos, presumably because of the associated reduction in lysosomal mannose 6-phosphate related intra-cellular transport (Fig.6A and B).

**Figure 7:**
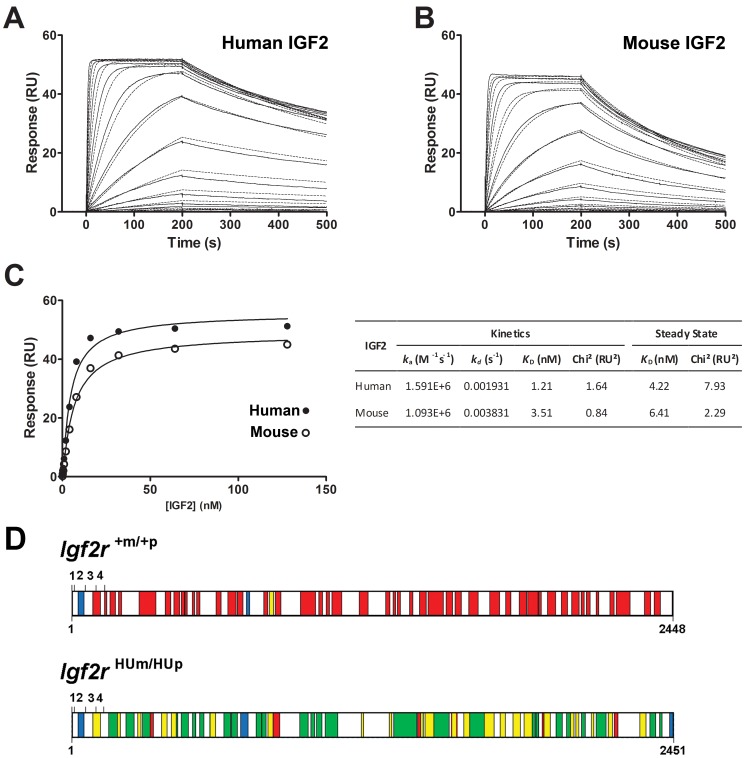
Surface Plasmon resonance binding kinetics of IGF2 binding to human IGF2R, and mass spectrometry (LCMS) of immune-precipitated liver IGF2R from control *Igf2r^+m/+p^* and homozygote transmission of *Igf2r^+HUm/HUp^*. Immobilised human IGF2R on a biosensor chip were bound to either recombinant human (A) or mouse (B) IGF2 at increasing concentrations, and real time kinetics fitted to a 1∶1 binding model. (C) steady state binding kinetics were also calculated for both interactions and tabulated. Mouse IGF2 has slightly lower K_D_ (nM). (D) peptides identified by mass spectrometry in samples of immuno-precipitated *Igf2r^+m/+p^* (WT) and *Igf2r^HUm/HUp^*. In both samples, red boxes depict unique peptides matching mouse IGF2R protein sequence, green boxes depict unique peptides matching human IGF2R protein sequence, and yellow boxes depict areas of overlap between mouse and human peptides. Blue boxes depict peptides common to both sequences. Exons 1–4 are represented by upper numbers, lower numbers represent amino acid residue number. Both mouse and human peptides were detectable in suggesting trans-splicing and generation of mouse and humanised proteins from the humanised alleles.

It was possible that the humanised allele associated overgrowth may have also related to changes in relative affinity of human IGF2R to mouse IGF2. In order to address this possibility, we expressed and purified the extra-cellular domains (domains 1–15) of recombinant human IGF2R and performed surface plasmon resonance to determine relative affinities to human and mouse IGF2. We show that the affinity (K_D_) of recombinant human IGF2R for mouse IGF2 is high between 3–6 nM, but slightly lower than that of human IGF2 (1–4 nM) (Fig. 7A, B and C).

In relation to the detection of mouse transcripts in homozygote *Igf2r^HUm/HUp^*, we performed mass spectrometry using immune-precipitated IGF2R from *Igf2r^HUm/HUp^* and *Igf2r^+m/+p^* control livers in order to circumvent the possibility of a PCR artefact. Screening of the peptides revealed that both human and mouse peptides could be easily detected in the humanised sample, meaning that the mouse allele was being co-expressed with the humanised allele (Fig. 7, Table S1). The Exponentially Modified Protein Abundance Index (emPAI) offers an approximate relative quantification of the proteins in a mixture based on protein coverage by the peptide matches. This value was 5.65 for the humanised protein and 0.64 for the mouse protein derived from the homozygous humanised mouse, confirming the presence of both proteins and indicating a relative excess of the humanised to mouse protein [29]. The overall efficiency of IGf2R protein expression was clearly impaired as total protein levels were low, and we assume this was due to disruption of splicing from the human exon 3–48 cDNA to the endogenous mouse wild-type exons. We conclude that the introduction of the construct in intron 2 not only disrupted imprinting control region (*Airn*) expression but may have also impaired splicing efficiency and mRNA production.

## Discussion

Gene dosage effects are a feature of imprinted, mono-allelic expressing and tumour suppressor genes. Gain of function, through bi-allelic *Igf2r* expression, and complete loss of function, through disruption of the maternal expressed allele, have been studied in the mouse. For the former, the assumption is that the extra-supply of the wild-type IGF2R protein does not saturate pathways, generate un-physiological effects or induce feedback inhibition. Moreover, complete loss of the protein following gene disruption can result in destabilisation of normally associated protein– protein complexes and result in adaptation that may misrepresent or mask the true function of the protein. It remains the case that comprehensive analysis of gene function that includes hypomorphic alleles have been poorly studied in the mouse, at a time when our knowledge is expanding in humans of the potential effects of functional titration either through modifier alleles or via variants in gene expression and protein supply that are frequently correlated with disease.

For the pathways regulated by *Igf2*, hypomorphic alleles have been studied in most depth in *C. elegan*s and *D. melanogaster* [30], [31], [32]. In the mouse, haplo-insufficiency and hypomorphic alleles of *Igf1r* have been most studied in relation to aging [33], [34], [35], [36]. PTEN is also an example one of the downstream IGF signalling proteins that acts to dephosphorylate phosphatidyl inositol (3,4,5) triphosphate to the diphosphate (4,5), thus reducing activation of Akt driven by the IGF2 ligand. Heterozygous *Pten* null mice develop lymphoid, prostate, breast, endometrial and intestinal neoplasms that mimic human Cowden syndrome, with hypomorphic alleles resulting in the development of prostate cancer [37]. Moreover, *Pten* function can be titrated by increased expression of a *Pten* pseudogene that sequesters regulatory miRNA, and even these subtle hypermorphic alleles can also titrate against tumour formation [38], [39]. Additive effects of *Pten* and *Igf2* in pathway activation can also be modified, for example following titration of *Igf2* dosage in *Pten* heterozygotes, resulting in corresponding titration of embryonic, placental and tumour phenotypes [40].

Unlike *Igf2,* the physiological variants that may modify *Igf2r* remains poorly understood. For example, *Igf2r* is resistant to imprinting de-regulation following the effects of gain of function and hypomorphic alleles of DNA methyl-transferases [41], . The lethality phenotype of *Igf2r^−m/+p^* also appears strain dependent, but it remains unclear what the functional mechanisms are with respect to associated modifier loci [44]. Although synonymous and non-synonymous polymorphisms are known in human, there remains some debate as to whether they have direct functional effects on IGF2R [24], [26], [45], 46,47,48,49,50. For example, structure and function studies of the IGF2R exon 34 (5002A>G, Gly1619Arg) polymorphism (rs629849) failed to detect any functional effects on domain 11, the IGF2 binding domain on IGF2R [26]. To date there have been no polymorphic human variants described relating to splicing regulation and post-translational processing.

Loss of function of *Igf2r* maternal expressed allele is known to result in peri-natal lethality thought due to cardiac enlargement and oedema [12], [13], [14]. The implications of these findings also suggest that loss of mannose 6-phosphate binding and lysosomal enzyme supply may contribute to the disproportionate growth phenotype, as the mechanism of growth regulation of *Igf2* appears proportionate on the mechanisms of cell proliferation and cell death at embryonic day 9 [51]. Through the insertion of a human cDNA that generated a humanised *Igf2r* allele, we wished to generate a system that we could easily modify, in order to investigate the *in vivo* function of human polymorphisms and mutations. By disrupting splicing regulation of both the targeted allele and the endogenous mouse *Igf2r* allele, we generated a reduction in overall IGF2R protein levels. We confirmed that the peri-natal lethality was dependent on *Igf2*, and we showed that we could rescue the phenotype by expression of a wild-type endogenous *Igf2r* allele. Interestingly, our experiments also suggest an effect associated with disruption of intron 2 *Airn* expression as a result of insertion of the construct. This site of insertion is known to alter the levels of *Airn* long non-coding RNA implicated in imprinting regulation of *Igf2r* expression, a long non-coding though to act in ‘cis’. Importantly, in our case the loss of *Igf2* dependent growth control function of the humanised *Igf2r* allele may have unmasked previously undetected effects. Our data now suggest that *Airn* may be acting in ‘trans’ to modify the expression of the paternal transmitted wild-type allele during spermatogenesis. However, these observations require further investigation.

The consequence of the humanised knock-in of a wild-type human cDNA was the generation of a hypomorphic allele of *Igf2r* with ∼50% of the functional effectiveness of a endogenous mouse wild-type allele. We showed that the affinity (K_D_) of recombinant human IGF2R for mouse IGF2 was high between 3–6 nM, but slightly lower than that of human IGF2 (1–4 nM). Compared to previously determined IGF2R:IGF2 affinities, the alteration in affinity as a result of humanisation is unlikely to have significant effects on overall IGF2 binding functions. We suggest that the internalisation rate of the receptor is likely to have a major role, implicating overall protein abundance as a major contributor to IGF2 clearance in context of such high relative affinities [27]. However, we have not determined the affinity of mouse IGF2 for mouse IGF2R, and it may be that this overall affinity is closer to that detected with recombinant human proteins. Thus, the main reason that accounted for the hypomorphic function of the humanised allele was the lack of expression of a fully spliced mRNA, resulting in reduced overall transcript levels and secondary reduction in protein supply. The quantification of mRNA correlated with levels of protein of the correct molecular weight, findings that were confirmed using mass spectrometry. These data suggest that *Igf2r* has an essential developmental role in IGF2 regulation, and that a single expressed wild-type allele of *Igf2r* is not providing IGF2R in excess, but it may be just enough to sequester IGF2 and prevent disproportionate growth and lethality. As a result, it is also likely that hypomorphic alleles are not expected to be evolutionary selected if they result in less than 50% of the functional activity of the wild-type allele.

## Materials and Methods

### Mouse Generation

Genetic targeting of *Igf2r* to insert a humanised allele was carried out in collaboration with Artemis Taconic (Cologne, Germany) as described in Figure 1. Viable mice were generated bearing the humanised allele on the paternal allele (*Igf2r* ^+*m/HUp*^). Subsequent animal work was approved by University of Oxford animal ethics committee and UK Home Office Project Licence.

The pIgf2r targeting vector was constructed using a PCR based cloning strategy. The flanking short arm (3.9 Kb) containing exons 3 and 4, and the long arm (7.6 Kb) containing exon 2, were generated using BAC clones from the C57BL/6J RPCI-23 BAC library. The human *IGF2R* mini-gene was derived from a human placenta cDNA (ATCC) as previously described [26]. The mini-gene consisted the human *IGF2R* sequence from the beginning of exon 3 to the STOP codon in exon 48. The human exon 3–48 cDNA was sequenced to ensure there were no PCR related mutations, and engineered at the 5′ site to incorporate the natural splice acceptor site located in front of exon 3 in the endogenous mouse locus as well as at the 3′ site to incorporate the mouse 3′ un-translated region (Fig. 1). The positive selection marker (PuroR) was flanked by loxP sites and inserted downstream of the IGF2R minigene. The targeting vector was then linearised with *Not*I, as shown in Fig. 1A.

The C57BL/6N Tac ES cell line was grown on a mitotically inactivated feeder layer comprised of mouse embryonic fibroblasts (MEF) in DMEM High Glucose medium containing 20% FBS (PAN) and 1200u per mL Leukemia Inhibitory Factor (Millipore ESG 1107). 1×10^7^ cells and 30 µg of linearised DNA vector was electroporated (Biorad Gene Pulser) at 240V and 200 µF. Puromycin selection (1 µg ml*^−^* ^1^) started on d2, counter-selection with gancyclovir (2 µM) started on d5 after electroporation. ES clones were isolated on d8 and analysed by Southern blotting according to standard procedures after expansion and freezing of clones in liquid nitrogen, as shown (Fig. 1B).

Primer sequence of 5e1 probe: sense: ATACTGGCTGAGCGATGTCCTTAGC antisense: CAACTCATCATACACCCTTG. Primer sequence of 3e1 probe: sense:TGTGATGATGTAATGGGCGAGG antisense: GGGTCACCAAGTGACCTGGGTC. Super-ovulated Balb/c females were mated with Balb/c males. Blastocysts were isolated from the uterus at dpc3.5. For microinjection, blastocysts were placed in a drop of DMEM with 15% FCS under mineral oil. A flat tip, piezo actuated microinjection-pipette with an internal diameter of 12–15 micrometer was used to inject 10–15 targeted C57BL/6N Tac ES cells into each blastocyst. After recovery, 8 injected blastocysts were transferred to each uterine horn of 2.5dpc pseudo-pregnant NMRI females. Chimerism was measured in chimeras (GO) by coat colour contribution of ES cells to the Balb/c host (black/white). Highly chimeric mice were bred to Cre-deletor C57BL/6 females in order to induce removal of the selection marker. Germline transmission was identified by the presence of black, strain C57BL/6 offspring (G1) and confirmed by PCR genotyping (Fig. 1C) using the following primers:

Igf2r_oligo1 = GGTTTATGCCTGATTTTGCTAGC.

Igf2r_oligo2 = TTCCACGCGTTAGAGGATTCC.

Igf2r_oligo3 = CCTCGTGTAGTTCAGAACACTGG.

Igf2r_oligo4 = GGTGAGGGTTCCACTGATCC.

### Mouse Breeding

Two female and two male *Igf2r* ^+m/HUp^ lines were paired with C57BL/6J wild-type mice and the resulting offspring genotyped. *Igf2r^+m/HUp^* males and females from line 2 (A-D09) were paired with wild-type C57BL/6J mice for all breeding apart from the heterozygote or homozygote inter-crosses, *R2Δ^−m/−p^* and *Igf2^+m/−p^* mice were obtained with permission and crossed with *Igf2r^+m/HUp^* [7], [52] animals. Embryos were staged by taking mid-day on the day of seminal plug detection as embryonic day 0.5 (E0.5). Weighed embryos were fixed in 4% neutral buffered formalin.

### PCR Genotyping to Determine Paternal Origin

Genotyping was performed by RT- PCR using standard conditions as above. Primers were as follows (5′–3′): Mouse exon 1; GCCCAGGCCGTCGACTTGGACGCC, as the forward primer, and mouse exon 3; GTCTGGATTCTGTGCTGTGAATCTGAAGGC, and human exon 3; CTCTGGACTCTGTGATTTGTGCCTTGC, as the reverse primers for the humanised and mouse alleles, respectively. PCR products were resolved on agarose gels.

### Protein Extraction and Western Blotting

The corresponding tissue was homogenised in ice-cold RIPA buffer (50 mM Tris-HCl pH 8.0, 150 mM NaCl, 1% Triton X-100, 0.5% sodium deoxycholate, 0.1% SDS) in the presence of 2x protease-phosphatase inhibitor cocktail (Halt cocktail, Pierce). The homogenates were incubated with rotation at 4°C for 2h and the supernatants were collected after centrifugation at 13,000 rpm at 4°C for 30 minutes and stored at −80°C. Total protein concentration was determined using the CB-X Protein Assay (G-Biosciences). 20 µg of total protein (mixed with an equal amount of 2x SDS loading buffer (Sigma) and heated at 95°C for 5 mins) were separated by glycine or tricine (for detection of IGF2) SDS-PAGE and subsequently transferred to PVDF membrane (Immobilon P, Millipore). Membranes were blocked in 5% milk in TBS-Tween 20 for 1h. The membranes were then incubated overnight at 4°C with primary antibodies in blocking buffer containing 5% milk. Antibodies used were anti-IGF2 (R&D systems, AF792) at 0.4 µg ml*^−^* ^1^, anti-IGF2R (R&D systems, AF2447) at 0.1 µg ml*^−^* ^1^, anti-Cathepsin L (R&D systems, AF1515) at 0.1 µg ml*^−^* ^1^. After extensive washing, the membranes were then incubated with the corresponding HRP-conjugated secondary antibody (Dako, 1∶2000) for 1h at room temperature in blocking buffer. The signals were visualized by the enhanced chemiluminescence system (EZ-ECL, Biological Industries). Densitometry was performed using ImageJ (NIH) and was normalized to β-actin or α-tubulin.

### Quantitative RT-PCR Analysis

RNA was isolated from individual limb tissue from embryos at E18.5 using TRI reagent following the manufacturer’s instructions (Applied Biosystems). Possible contaminant genomic DNA was removed using the TURBO DNA-free kit (Applied Biosystems). cDNA was synthesized from 1.5 µg of total RNA using the High Capacity cDNA Reverse Transcription Kit (Applied Biosystems). Real-time PCR (RT-PCR) analysis was performed in Rotor Gene Q PCR cycler (Qiagene). A standard curve was performed with serial dilutions of cDNA. Experiments were run in duplicate and expression levels were normalized to GAPDH expression levels using qGene software. Primer sequences used to amplify cDNA fragments were (5′–3′): human *Igf2r*: TGGCCCTGTTGCTCTACAA and AGCAAGTGGTCAGCTTACTTATC; mouse *Igf2r*: CCTTCTCTAGTGGATTGTCAAGTG and AGGGCGCTCAAGTCATACT; *gapdh*: CAATGAATACGGCTACAGCAAC and TTACTCCTTGGAGGCCATGT. The following probes from Roche Universal Probe Library were used: 77 (mouse) for Gapdh, 33 (mouse) and 55 (human) for Igf2r. The mouse primers amplify a fragment that covers the end of exon 23 and the beginning of exon 24, and the human primers amplify a fragment covering the end of exon 46 and the beginning of exon 47.

### Tissue Imaging

Embryos were fixed in 4% neutral buffered formalin at RT for 24h before processing and paraffin embedding. 5 µm sections were cut using a microtome, and H&E staining performed by standard techniques. Slides were de-waxed in xylene, rehydrated through a graded alcohol series followed by antigen de-masking in sodium citrate buffer (10 mM, pH 6.0) in a pressure cooker for 2 min at 125°C and 10 min at 85°C. After 3 washes in 1x Tris-buffered saline (TBS, pH7.4 tissue sections were blocked in 10% donkey serum/Tween 20^©^ 0.5% (v/v) for 1 h at RT followed by simultaneous incubation with primary antibodies anti-IGF2R (goat, 1∶200, AF2447, R&D Systems) and anti-LAMP1 (rat, 1∶100, sc19992, Santa Cruz) at 4°C overnight. After 3 washes in TBS tissue sections were incubated with secondary antibodies donkey anti-goat Alexa488 and donkey anti-rat Alexa594 (1∶300, Invitrogen) simultaneously for 2 h at RT. After washing tissue sections were counterstained with DAPI (1∶5000, Invitrogen) and mounted with Prolong anti-fade (Invitrogen). Fluorescent images were acquired using a confocal microscope Olympus Fluoview FV1000 with each image having a size of 2048×2048 pixels. Images of Haematoxylin & Eosin (H&E) sections were acquired by an Olympus microscope BX60 in combination with Nuance CCD system and software.

### Morphometric Analysis of Heart Development

A comparative morphometric analysis of heart development in wild-type (*Igf2r^+m/+p^*, n = 3) and heterozygous mice (*Igf2r^HUm/+p^*, n = 4) was performed using Image J. Five transverse, consecutive H&E sections of the central heart area encompassing ventricles and atria-ventricular valves of each mouse were assessed in regard to the heart area, heart muscle area, blood filled spaces and nuclear area. The heart was identified as a region of interest, prior to thresholding and Otsu automated segmentation methods to determine areas measured in pixels. Ratios of areas using were determined for each section and were compared using students t-test.

### Surface Plasmon Resonance

The 1–15 extracellular domains of human IGF2R were expressed in HEK293T cells as CD4 (domains 3 and 4) fusion protein cloned in the pHLsecAvitag [53]. The cells were co-transfected with a plasmid for biotin ligase BirA expression and the medium was supplemented with 2 mM biotin. The biotinylated protein was purified using a PrepEase His-tagged protein purification midi kit (USB Affimetrix, UK).

Surface plasmon resonance analysis of human and mouse IGF2 binding to human IGF2R was performed on a BIAcore T200. 1800 response units of biotinylated IGF2R fusion protein was immobilized on a CM5 chip previously loaded with Streptavidin by amine coupling. Kinetic binding experiments were carried out at 25°C at a 40 µL/min flow rate in HBS-EP binding buffer by injecting solutions of IGF2 from both species ranging from 256 to 0.125 nM for 200 seconds. Analyte solutions were then replaced by HBS-EP buffer for 1 h and followed by a 60 µL injection of 2 M MgCl_2_ for regeneration of the sensor chip surface. A buffer control and a reference flow cell were included. Data were analysed using the BIAcore T200 Evaluation software version 1.0 and both by fitting the kinetic data to 1∶1 binding model and by steady state analysis of the data reaching equilibrium.

### Immuno-precipitation and Mass Spectrometry Analysis

Immuno-precipitations (IPs) were performed from adult WT or homozygous *Igf2r^HUm/HUp^* mouse liver lysates using the Protein G immuno-precipitation kit (Roche) according to the manufacturer’s instructions. A total of 300 µg of protein were incubated with 1 µg of an anti-IGF2R antibody (R&D systems, AF2447). Immuno-precipitated proteins were separated on an SDS-PAGE gel (4%–12% NuPage), stained with Coomassie Blue, and the band corresponding to IGF2R was cut into 1 mm^3^ cubes. The band was reduced, alkylated, digested with trypsin according to standard proteomics practices [54], and the resulting peptides were analyzed by LCMS on an Orbitrap (ThermoFisher) mass spectrometer as described [55]. Database searching was performed by Mascot against the REFSEQ database of mouse proteins as of June 2006, including the humanised sequence of *Igf2r*.

## Supporting Information

Table S1 **Mass spectrometry identification of the full size IGF2R species present in both Igf2r+m/+p and Igf2rHUm/HUp mice. Sequence of the peptides matching mouse IGF2R (Q07113) or humanised IGF2R (hybrid sequence consisting of residues exon 1 to 2 from Q07113 followed by exons 3–48 from P11717). Peptides shown have rank = 1 and score>25.**
(DOCX)
