# Supplementary material for: Maternal Transmission of a Humanised Igf2r Allele Results in an Igf2 Dependent Hypomorphic and Non-Viable Growth Phenotype
Source: PLoS One. 2013 Feb 28;8(2):e57270. doi: 10.1371/journal.pone.0057270 (PMC3585325; doi:10.1371/journal.pone.0057270)
Supplement: Table S1 — Mass spectrometry identification of the full size IGF2R species present in both Igf2r+m/+p and Igf2rHUm/HUp mice. Sequence of the peptides matching mouse IGF2R (Q07113) or humanised IGF2R (hybrid sequence consisting of residues exon 1 to 2 from Q07113 followed by exons 3–48 from P11717). Peptides shown have rank = 1 and score>25. (DOCX) [file pone.0057270.s001.docx]

**Supplementary Information**

**Maternal transmission of a humanised *Igf2r* allele results in an *Igf2* dependent hypomorphic and non-viable growth phenotype**.

Jennifer Hughes*, Susana Frago*, Claudia Bühnemann, Emma J. Carter, A. Bassim Hassan^¶^

**Legend**

**Table 1.**

Mass spectrometry identification of the full size IGF2R species present in both *Igf2r^+m/+p^* and *Igf2r^HUm/HUp^* mice. Sequence of the peptides matching mouse IGF2R (Q07113) or humanised IGF2R (hybrid sequence consisting of residues exon 1 to 2 from Q07113 followed by exons 3-48 from P11717). Peptides shown have rank=1 and score>25.

**Table 1.**

| Igf2r ^+m/+p^ mouse sample |  |  |  |  | |
| --- | --- | --- | --- | --- | --- |
| Unique peptides matching mouse IGF2R (Q07113) |  | **Unique peptides matching humanised IGF2R** |  | **Peptides matching both IGF2R sequences** | |
| Peptide sequence | **Score** | **Peptide sequence** | **Score** | **Peptide sequence** | **Score** |
|  |  |  |  |  |  |
| IQTSITFLCGK | 48 | YYINVCRPLNPVPGCNR | 35 | INVCGNVGISSCGPTSAICMCDLK | 70 |
| TLGTPEFVTATDCVHYFEWR | 28 |  |  | ATLITFLCDR | 71 |
| EVPCYAFDDK | 38 |  |  |  |  |
| LNGGYLVDDSDPDTSLFINVCR | 110 |  |  |  |  |
| VCPAGTAACLLK | 33 |  |  |  |  |
| YEVEWITEYACHR | 82 |  |  |  |  |
| DYLQSESCSLSSEQHDITIDLSPLAQYGGSPYVSDGR | 136 |  |  |  |  |
| EYTFFINVCGDTK | 60 |  |  |  |  |
| YSDGDLTLIYSGGDECSSGFQR | 94 |  |  |  |  |
| DGRGEPVFTGEVDCTYFFTWDTK | 34 |  |  |  |  |
| EDLLCGAINGK | 91 |  |  |  |  |
| YDLSVLAR | 49 |  |  |  |  |
| YFFINVCHR | 29 |  |  |  |  |
| NCPEDAAVCAVDK | 64 |  |  |  |  |
| SDGCFYEFEWHTAAACVLSK | 54 |  |  |  |  |
| VETEKYDFYINVCGPVSMDPCQSNSGACQVAK | 69 |  |  |  |  |
| SWNLGLSSTK | 35 |  |  |  |  |
| LTYYDGMIQLSYR | 87 |  |  |  |  |
| SEGGSGGNWYAMENSR | 84 |  |  |  |  |
| YENHEGSLAETVSISNLGVAK | 84 |  |  |  |  |
| DPSSGFVFNLSPLNDSAQGHVVLGIGK | 67 |  |  |  |  |
| TFVFNICGAMPACGTVAGKPAYGCEAETQIEDIK | 59 |  |  |  |  |
| SLQLSAEGFLTLTYK | 99 |  |  |  |  |
| FICNDDIYPGAPK | 37 |  |  |  |  |
| NTYFEFETALACTPSLVDCQVTDPAGNEYDLSALSMVR | 61 |  |  |  |  |
| IVFECAQTSGSPMFQFVNNCEYVFVWR | 26 |  |  |  |  |
| TVEACPVIR | 51 |  |  |  |  |
| VAGLLSQK | 31 |  |  |  |  |
| LTFENGLLK | 44 |  |  |  |  |
| STTIYFYCDR | 47 |  |  |  |  |
| ETSDCSYMFEWR | 74 |  |  |  |  |
| TGATEHYLINVCK | 27 |  |  |  |  |
| VRDGPQWTDGVTVLQYVDGDLCPDK | 74 |  |  |  |  |
| DGPQWTDGVTVLQYVDGDLCPDK | 96 |  |  |  |  |
| DGPQWTDGVTVLQYVDGDLCPDKIR | 92 |  |  |  |  |
| FTCSDNQVNSRPLFISAVQDCEYTFSWPTPSACPVK | 48 |  |  |  |  |
| SNTHDDCQVTNPSTGHLFDLSSLSGR | 55 |  |  |  |  |
| GLVFMSICEENENCGPGVGACFGQTR | 94 |  |  |  |  |
| LSYKDQVLQLVYENGSPCPSLSDLR | 74 |  |  |  |  |
| QTCTLFFSWHTPLACEQATECTVR | 57 |  |  |  |  |
| NGSSIIDLSPLIHR | 42 |  |  |  |  |
| VPVDGPPIDIGR | 93 |  |  |  |  |
| VTGPPIFNPVANEVYLNFESSTHCLADR | 77 |  |  |  |  |
| TNDCDFVFEWETPIVCPDEVK | 75 |  |  |  |  |
| TYSIGVCTAAAGLGQEGCK | 79 |  |  |  |  |
| DGGVCLLSGNK | 75 |  |  |  |  |
| LASMQLDYR | 63 |  |  |  |  |
| HQDEAVILSYVNGDPCPPETDDGEPCVFPFIYK | 91 |  |  |  |  |
| SYDECVLEGR | 31 |  |  |  |  |
| MSAIIFTCDESEDIGRPQVFSEDR | 108 |  |  |  |  |
| GCEVTFEWK | 63 |  |  |  |  |
| LLSSLTGSWDFVHEGNSYFINLCQR | 71 |  |  |  |  |
| SATGQVQVLGLVHTQK | 79 |  |  |  |  |
| LEVIDETVIVTYSK | 109 |  |  |  |  |
| TASSVIELTCAK | 61 |  |  |  |  |
| FDSVSCTYYFYWYSR | 58 |  |  |  |  |
| SFSLGEIYFK | 54 |  |  |  |  |
| YYVQDGDLDVVFTSSSK | 63 |  |  |  |  |
| SVSSTIFFHCDPLVK | 55 |  |  |  |  |
| DGIPEFSHETADCQYLFSWYTSAVCPLGVDFEDESAGPEYK | 32 |  |  |  |  |
| VSKEEETDENETEWLMEEIQVPAPR | 48 |  |  |  |  |
| AEALSSLHGDDQDSEDEVLTVPEVK | 36 |  |  |  |  |
| Igf2r^Hu m/ Hu p^ mouse sample |  |  |  |  |  |
| Unique peptides matching humanised IGF2R |  | **Unique peptides matching mouse IGF2R (Q07113)** |  | **Peptides matching both IGF2R sequences** |  |
| Peptide sequence | **Score** | **Peptide sequence** | **Score** | **Peptide sequence** | **Score** |
|  |  |  |  |  |  |
| VQSSIAFLCGK | 65 | IQTSITFLCGK | 49 | INVCGNVGISSCGPTSAICMCDLK | 97 |
| TLGTPEFVTATECVHYFEWR | 78 | TLGTPEFVTATECVHYFEWR | 137 | ATLITFLCDR | 68 |
| LSGAYLVDDSDPDTSLFINVCR | 126 | VCPAGTAACLLK | 28 | DAGVGFPEYQEEDNSTYNFR | 100 |
| DIDTLRDPGSQLR | 27 | YEVEWITEYACHR | 50 |  |  |
| ACPPGTAACLVR | 38 | EYTFFINVCGDTK | 68 |  |  |
| LVLSYVR | 39 | YSDGDLTLIYSGGDECSSGFQR | 83 |  |  |
| EEAGKLDFCDGHSPAVTITFVCPSER | 75 | GEPVFTGEVDCTYFFTWDTK | 102 |  |  |
| YEIEWITEYACHR | 88 | SDGCFYEFEWHTAAACVLSK | 56 |  |  |
| TCSLSGEQQDVSIDLTPLAQSGGSSYISDGK | 66 | YENHEGSLAETVSISNLGVAK | 49 |  |  |
| YSDGDLTLIYFGGDECSSGFQR | 117 | TVEACPVIR | 26 |  |  |
| GTPVFTGEVDCTYFFTWDTEYACVK | 70 | STTIYFYCDR | 55 |  |  |
| RYDLSALVR | 31 | DGPQWTDGVTVLQYVDGDLCPDK | 108 |  |  |
| HAEPEQNWEAVDGSQTETEK | 32 | DGPQWTDGVTVLQYVDGDLCPDKIR | 32 |  |  |
| GCPEDAAVCAVDK | 82 | FTCSDNQVNSRPLFISAVQDCEYTFSWPTPSACPVK | 40 |  |  |
| GNIQLSYSDGDDCGHGK | 39 | LSYKDQVLQLVYENGSPCPSLSDLR | 41 |  |  |
| TSGEGGCFYEFEWHTAAACVLSK | 93 | QTCTLFFSWHTPLACEQATECTVR | 50 |  |  |
| KYDFYINVCGPVSVSPCQPDSGACQVAK | 94 | VPVDGPPIDIGR | 70 |  |  |
| TWNLGLSNAK | 46 | VTGPPIFNPVANEVYLNFESSTHCLADR | 32 |  |  |
| LSYYDGMIQLNYR | 84 | TNDCDFVFEWETPIVCPDEVK | 108 |  |  |
| SEGGLGGNWYAMDNSGEHVTWR | 85 | TYSIGVCTAAAGLGQEGCK | 90 |  |  |
| YYINVCRPLNPVPGCNR | 63 | DGGVCLLSGNK | 52 |  |  |
| YEKDQGSFTEVVSISNLGMAK | 140 | HQDEAVILSYVNGDPCPPETDDGEPCVFPFIYK | 47 |  |  |
| TGPVVEDSGSLLLEYVNGSACTTSDGR | 68 | MSAIIFTCDESEDIGRPQVFSEDR | 62 |  |  |
| IFMFNVCGTMPVCGTILGKPASGCEAETQTEELK | 60 | GCEVTFEWK | 30 |  |  |
| SLQLSTEGFITLTYK | 94 | LEVIDETVIVTYSK | 86 |  |  |
| GTADAFIVR | 74 | TASSVIELTCAK | 48 |  |  |
| FVCNDDVYSGPLKFLHQD | 56 | SFSLGEIYFK | 54 |  |  |
| NTYFEFETALACVPSPVDCQVTDLAGNEYDLTGLSTVR | 30 | YYVQDGDLDVVFTSSSK | 109 |  |  |
| KPWTAVDTSVDGR | 65 | SVSSTIFFHCDPLVK | 32 |  |  |
| TVEACPVVR | 35 | VSKEEETDENETEWLMEEIQVPAPR | 39 |  |  |
| STAIFFYCDR | 57 |  |  |  |  |
| ETSDCSYLFEWR | 64 |  |  |  |  |
| TQYACPPFDLTECSFK | 96 |  |  |  |  |
| DGAGNSFDLSSLSRY | 102 |  |  |  |  |
| YSDNWEAITGTGDPEHYLINVCK | 96 |  |  |  |  |
| SLAPQAGTEPCPPEAAACLLGGSKPVNLGR | 59 |  |  |  |  |
| YVDGDLCPDGIR | 48 |  |  |  |  |
| FTCSESQVNSRPMFISAVEDCEYTFAWPTATACPMK | 57 |  |  |  |  |
| SNEHDDCQVTNPSTGHLFDLSSLSGR | 111 |  |  |  |  |
| AGFTAAYSEK | 57 |  |  |  |  |
| GLVYMSICGENENCPPGVGACFGQTR | 113 |  |  |  |  |
| YVDQVLQLVYKDGSPCPSK | 40 |  |  |  |  |
| QTCTLFFSWHTPLACEQATECSVR | 60 |  |  |  |  |
| NGSSIVDLSPLIHR | 63 |  |  |  |  |
| TGGYEAYDESEDDASDTNPDFYINICQPLNPMHGVPCPAGAAVCK | 117 |  |  |  |  |
| VPIDGPPIDIGR | 80 |  |  |  |  |
| VAGPPILNPIANEIYLNFESSTPCLADK | 42 |  |  |  |  |
| TSECDFVFEWETPVVCPDEVR | 92 |  |  |  |  |
| TYSVGVCTFAVGPEQGGCK | 89 |  |  |  |  |
| DGGVCLLSGTK | 58 |  |  |  |  |
| TYSVGVCTFAVGPEQGGCKDGGVCLLSGTK | 64 |  |  |  |  |
| HQDEAVVLSYVNGDR | 137 |  |  |  |  |
| CPPETDDGVPCVFPFIFNGK | 77 |  |  |  |  |
| SYEECIIESR | 63 |  |  |  |  |
| LWCSTTADYDR | 45 |  |  |  |  |
| CDEDEDIGRPQVFSEVR | 48 |  |  |  |  |
| GCDVTFEWK | 64 |  |  |  |  |
| LLSSLTGSWSLVHNGVSYYINLCQK | 54 |  |  |  |  |
| TTTGDVQVLGLVHTQK | 114 |  |  |  |  |
| LGVIGDKVVVTYSK | 44 |  |  |  |  |
| TASSVIELTCTK | 77 |  |  |  |  |
| FDIDSCTYYFSWDSR | 75 |  |  |  |  |
| SFSLGDIYFK | 50 |  |  |  |  |
| TNGDNYLYEIQLSSITSSR | 113 |  |  |  |  |
| NPACSGANICQVKPNDQHFSR | 49 |  |  |  |  |
| YYLQDGDLDVVFASSSK | 106 |  |  |  |  |
| VNKEEETDENETEWLMEEIQLPPPR | 72 |  |  |  |  |
| ALSSLHGDDQDSEDEVLTIPEVK | 86 |  |  |  |  |
| NAQSNALQER | 32 |  |  |  |  |
